# Genome-Wide Analysis of Strictosidine Synthase-like Gene Family Revealed Their Response to Biotic/Abiotic Stress in Poplar

**DOI:** 10.3390/ijms241210117

**Published:** 2023-06-14

**Authors:** Ruiqi Wang, Wenna Zhao, Wenjing Yao, Yuting Wang, Tingbo Jiang, Huanzhen Liu

**Affiliations:** 1 State Key Laboratory of Tree Genetics and Breeding, Northeast Forestry University, Harbin 150040, China; yjswrq@outlook.com (R.W.); zwn9426@163.com (W.Z.); yaowenjing@njfu.edu.cn (W.Y.); wyt1513026188@163.com (Y.W.); 2 Co-Innovation Center for Sustainable Forestry in Southern China, Bamboo Research Institute, Nanjing Forestry University, 159 Longpan Road, Nanjing 210037, China

**Keywords:** Strictosidine synthase, SSL, STR, stress, family, *Populus trichocarpa*

## Abstract

The *strictosidine synthase-like* (*SSL*) gene family is a small plant immune-regulated gene family that plays a critical role in plant resistance to biotic/abiotic stresses. To date, very little has been reported on the *SSL* gene in plants. In this study, a total of thirteen *SSL*s genes were identified from poplar, and these were classified into four subgroups based on multiple sequence alignment and phylogenetic tree analysis, and members of the same subgroup were found to have similar gene structures and motifs. The results of the collinearity analysis showed that poplar *SSL*s had more collinear genes in the woody plants *Salix purpurea* and *Eucalyptus grandis*. The promoter analysis revealed that the promoter region of *PtrSSL*s contains a large number of biotic/abiotic stress response elements. Subsequently, we examined the expression patterns of *PtrSSL*s following drought, salt, and leaf blight stress, using RT-qPCR to validate the response of *PtrSSL*s to biotic/abiotic stresses. In addition, the prediction of transcription factor (TF) regulatory networks identified several TFs, such as *ATMYB46*, *ATMYB15*, *AGL20*, *STOP1*, *ATWRKY65*, and so on, that may be induced in the expression of *PtrSSL*s in response to adversity stress. In conclusion, this study provides a solid basis for a functional analysis of the *SSL* gene family in response to biotic/abiotic stresses in poplar.

## 1. Introduction

In nature, plants defend themselves against damaging biotic and abiotic factors through a variety of defense responses [1]. In general, the various defense responses of plants depend on a transcriptional regulatory network consisting of top-level transcription factors (TFs) and bottom-level structural genes [2,3,4]. To date, a wide range of plant stress-response TFs and structural genes have been identified from different plant species that are important for maintaining plant identity, adapting to environmental changes, and supporting plant growth [5,6,7,8,9,10,11]. The plant *strictosidine synthase-like* (*SSL*) genes are a kind of gene closely related to plant immune regulation; they share a similar extracellular structural domain (of ca. 400 aa in length) with the animal immune protein hemomucin [12]. The enzyme is responsible for combining geraniol diphosphate and tryptamine precursors in the cytosolic matrix to produce the intermediate compound strictosidine (STR). STR is a precursor compound for the biosynthesis of many monoterpene alkaloids, which play an important role in the defense mechanisms of plants, enhancing their resistance to biotic/abiotic stresses such as negative environments, parasites, fungi, and bacteria. The plant *SSL* gene has two characteristic structural domains, the Strictosidine synthase domain (PF03088) and the Strictosidine synthase-like N-terminal domain (PF20067). The Strictosidine synthase domain is directly related to STR synthesis and the Strictosidine synthase-like N-terminal has a six-bladed beta-propeller fold structure and have similar mechanistic features to strictosidine synthase domain.

Currently, our limited knowledge of the plant SSL family comes mainly from *Arabidopsis thaliana*, and the research shows that all classes of *AtSSL* genes respond to a variety of biotic and abiotic stresses [13]. In addition, *AtSSL4-7* was detected to synthesize STR and was induced by various phytohormones, such as salicylic acid, methyl jasmonate, ethylene, and so on [1,13]. In other plants, there are a few reports of *SSL* genes being involved in plant resistance processes. For example, in *Catharanthus roseus*, drought stress was found to induce the expression of the *STR* gene up to 5.6-fold, which, in turn, alleviated drought damage on the plant [14]. Tomato miR1916 overexpression lines exhibited reduced tolerance to drought, possibly due to the repressive effect of miR1916 on the target *STR* gene [15]. Chitooligosaccharides are the only basic amino oligosaccharides with positively charged cations in nature which can improve crop resistance and yield [16,17]. It has been proposed that chitooligosaccharides may enhance plant stress tolerance by inducing the expression of *secologanin synthase* (*SLS*), *STR*, *strictosidine glucosidase* (*SGD*), and other genes [18]. In *Solanum lycopersicum*, silencing of sly-miR1916 was found to lead to increased expression of the target genes *STR-2*, *UDP-glycosyltransferases* (*UGT*s), *late blight resistance protein homolog R1B-16*, *disease resistance protein RPP13-like*, and *MYB transcription factor* (*MYB12*), which, in turn, enhanced the resistance of tomato leaves to *Phytophthora infestans* and *Botrytis cinerea* [19]. Overall, there is growing evidence that members of the *SSL* family can be involved in plant resistance to stress. However, no systematic analysis of the expression patterns of poplar *SSL* family members in response to biotic/abiotic stresses has yet been carried out.

*Populus trichocarpa* was selected as a research material for this study; it is the model plant in woody plant research and has had its genome sequenced with a clear genetic background [20]. However, there is a lack of research on the *SSL* family in *P. trichocarpa*. We identified a total of 13 *SSL* genes from poplar, using bioinformatics methods, and performed a gene structure analysis, chromosomal localization, a collinearity analysis, and a promoter analysis based on their clear genetic background. In the meanwhile, we analyzed the expression pattern of *PtrSSL*s under biotic/abiotic stresses, using RT-qPCR. This study provides new information about the evolutionary relationships of the *PtrSSL* family, which serves as a reference for understanding the biological functions of *SSL*s in poplar.

## 2. Result

### 2.1. Phylogenetic and Structural Analysis of PtrSSL Family

**Figure 1:**
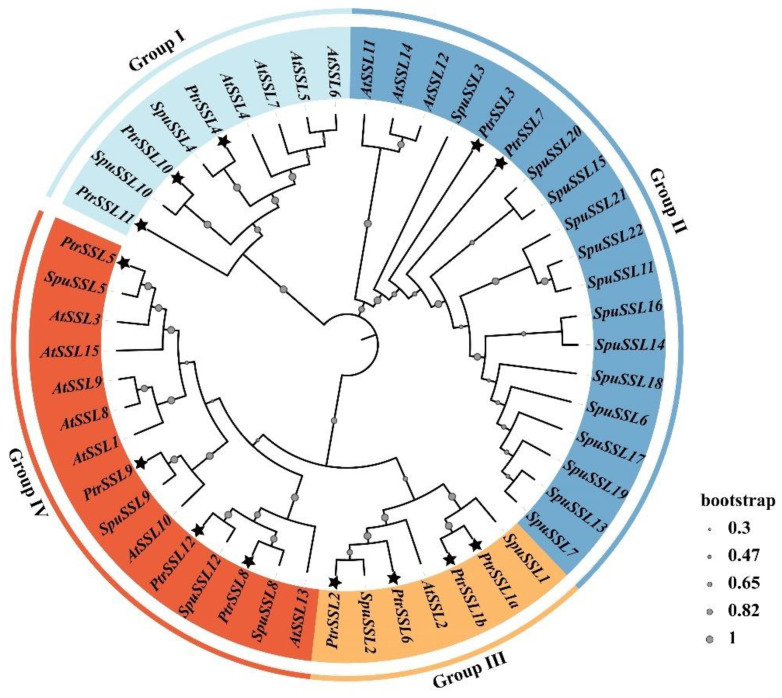
Phylogenetic analysis of *SSL*s in *Populus trichocarpa* (*Ptr*), *Arabidopsis thaliana* (*At*), and *Salix purpurea* (*Sup*). Neighbor-joining (NJ) method with 10,000 bootstrap replicates was applied to draw a phylogenetic tree by MEGA7 software. The tree was divided into four groups; each color represents one group. Black stars indicate *PtrSSL*s.

We used HMMER software to search for members of the *strictosidine synthase-like* (*SSL*) family in *Populus trichocarpa*, *Arabidopsis thaliana*, and *Salix purpurea* by mapping the conserved structural domains, PF03088 and PF20067, to protein sequences in the most recent version of the database for each species. Eventually, 13, 15, and 22 *SSL*s were obtained in *Populus trichocarpa*, *Arabidopsis thaliana*, and *Salix purpurea*, respectively, and phylogenetic trees were constructed. *SSL* family members of *Populus trichocarpa* and *Salix purpurea* were named regarding the previously published names of *Arabidopsis thaliana SSL*s, as shown in Figure 1 and Table 1. The phylogenetic analysis showed that the *SSL* family members could be divided into four groups, Groups I-IV, containing 9, 19, 7, and 15 *SSL*s, respectively (Figure 1). We found that each group contained *SSL*s from all three species, implying that *SSL*s from herbaceous and woody plants may have similar evolutionary patterns. The results also show that all branches of *PtrSSL*s are adjacent to branches of *SpuSSL*s compared to Arabidopsis, suggesting that the two woody species, *Populus trichocarpa* and *Salix purpurea*, are more closely related, and *SSL*s diverged with the evolution of the herbaceous woody plant.

Our analysis of the protein physicochemical properties showed that the length of the SSL family amino acids in all three species ranged from 98 (PtrSSL11) to 570 (PtrSSL4), and the molecular weight ranged from 10878.46 (PtrSSL11) to 62,550.88 (PtrSSL4). The pI of the SSL protein ranged from 4.69 (SpuSSL3) to 9.8 (SpuSSL20); a majority of AtSSL proteins (11/15) were acidic (pI < 7); and nearly half of PtrSSL and SpuSSL proteins were acidic, accounting for 6/13 and 11/22, respectively. The aliphatic index of the SSL protein ranged from 75.2 (AtSSL1) to 104.29 (AtSSL7), and most SSL proteins were stable. The grand average of hydropathicity (GRAVY) value ranged from −0.403 (AtSSL1) to 0.349 (SpuSSL3). Of these, 8/15 of the AtSSL proteins, 10/13 of the PtrSSL proteins, and 9/22 of the SpuSSL proteins were hydrophilic. Overall, the physicochemical properties of SSL proteins from different species are different (Table 1).

**Figure 2:**
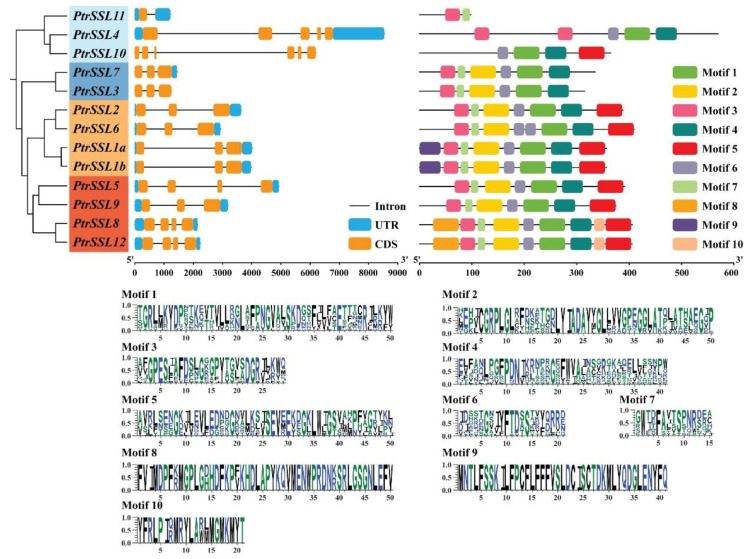
Gene structure and protein motif of the *SSL* gene family in poplar. Colorful boxes delineate different motifs. The clustering was performed according to the phylogenetic analysis.

In addition, we analyzed the gene structure and motif of *PtrSSL*s by using TBtools and MEME software. In general, the *SSL* genes that are in the same evolutionary branch have structural similarities. The genetic structure analysis revealed that Group I members contained 4–7 exons, Group II members contained 3–4 exons, all Group III members contained 5 exons, and Group IV members contained 5–6 exons (Figure 2). In Groups II–IV, the genetic structure of the members within each subgroup is similar, except for Group I, where the number of exons varies considerably between members. To further confirm the similar characteristics of the members of the subgroup, we performed a motif analysis, which revealed that the types and numbers of motifs contained in the members within each subgroup were similar in Groups II–IV (Figure 2). For example, the two members of Group II, *PtrSSL3* and *PtrSSL7*, have the same type and number of motifs. We also found that the motif distributions of Group III and Group IV were very similar, probably because the two subgroups originated from the same evolutionary branch. Only the three members of Group I differed significantly in the type of motif they contained, implying that the functions of the three genes may differ significantly. Overall, this corresponds to the results of our phylogenetic tree analysis.

### 2.2. Chromosomal Distribution and Collinearity Analysis of PtrSSLs

**Figure 3:**
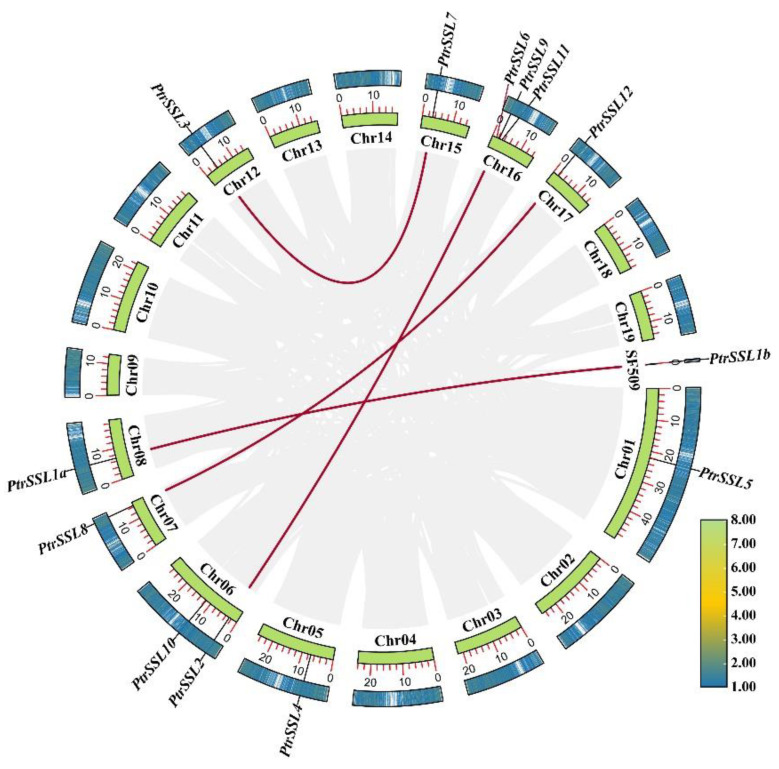
Chromosomal localization and collinearity analysis of the *PtrSSL* family in poplar. Heatmap in the outer circles indicate gene density on chromosomes, with red and gray lines indicating duplication gene pairs of *PtrSSL*s and collinear gene pairs in the poplar genome, respectively.

To explore the distribution of PtrSSLs on poplar chromosomes, we determined the chromosome location information of *PtrSSL*s according to the *Populus trichocarpa* v4.1 database in Phytozome (Supplementary Table S1). We also mapped the gene distribution based on their starting position on the chromosome (Figure 3). The results showed that there were 13 *PtrSSL*s not evenly distributed on the 9 chromosomes and 1 scaffold (Figure 3). Chr16 contains the greatest number of *PtrSSL*s, namely *PtrSSL*6, *PtrSSL*9, and *PtrSSL*11. Chr06 contains two *PtrSSL*s, namely *PtrSSL*2 and *PtrSSL*10. Chr01, Chr05, Chr07, Chr08, Chr12, Chr15, Chr17, and scaffold_509 each have one *PtrSSL* distributed as *PtrSSL5*, *PtrSSL4*, *PtrSSL8*, *PtrSSL1a*, *PtrSSL3*, *PtrSSL7*, *PtrSSL12*, and *PtrSSL1b*, respectively. To explore the gene duplication events of *PtrSSL* family members, a collinearity analysis was performed by using MCScanX. A total of four highly homologous gene pairs were obtained, both of which were segmental duplications (Figure 3). The Ka/Ks of these genes were both less than 1, indicating that a strong purifying selection was experienced (Table 2). All gene pairs are more than 80% homologous, indicating that they evolved to form paralogous genes as a result of gene duplication events. In addition, *PtrSSL1a* and *PtrSSL1b* have identical CDS and amino acid sequences, resulting in Ka and Ks values of 0. Although they are highly homologous, they originate from different chromosomes, Chr08 and scaffold 509, and are therefore judged to be two genes.

**Figure 4:**
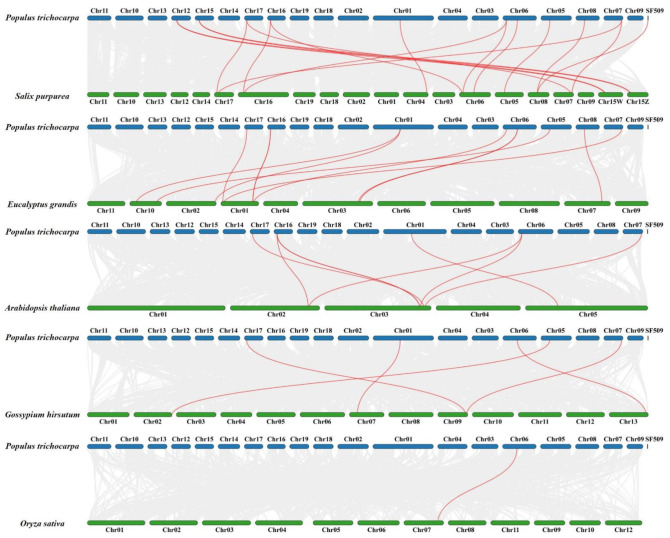
Collinearity analysis of the *SSL* genes from poplar and five other species. The *SSL* collinear genes are connected with a red line, while other collinear genes are connected with gray line.

To further investigate the evolutionary relationship of *PtrSSL*s, we constructed a phylogram of *SSL* genes between *P. trichocarpa* with five other plant species, including four dicots (*Salix purpurea*, *Eucalyptus grandis*, *Arabidopsis thaliana*, and *Gossypium hirsutum*) and one monocot (*Oryza sativa*). As shown in Figure 4 and Supplementary Table S2, there were 18, 11, 8, 5, and 1 homologous pair/s between *P. trichocarpa* with *S. purpurea*, *E. grandis*, *A. thaliana*, *G. hirsutum*, and *O. sativa*, respectively. Six *PtrSSL*s (*PtSSL2*, *PtSSL5*, *PtSSL6*, *PtSSL8*, *PtSSL10*, and *PtSSL12*) showed a high level of collinearity with other species *SSL* genes (collinear genes = 5). Overall, poplar *SSL* genes have the highest number of collinear genes, with those being in *S. purpurea* and *E. grandis*, which are also woody dicotyledons.

### 2.3. Cis-Elements Analysis of PtrSSLs Promoters

**Figure 5:**
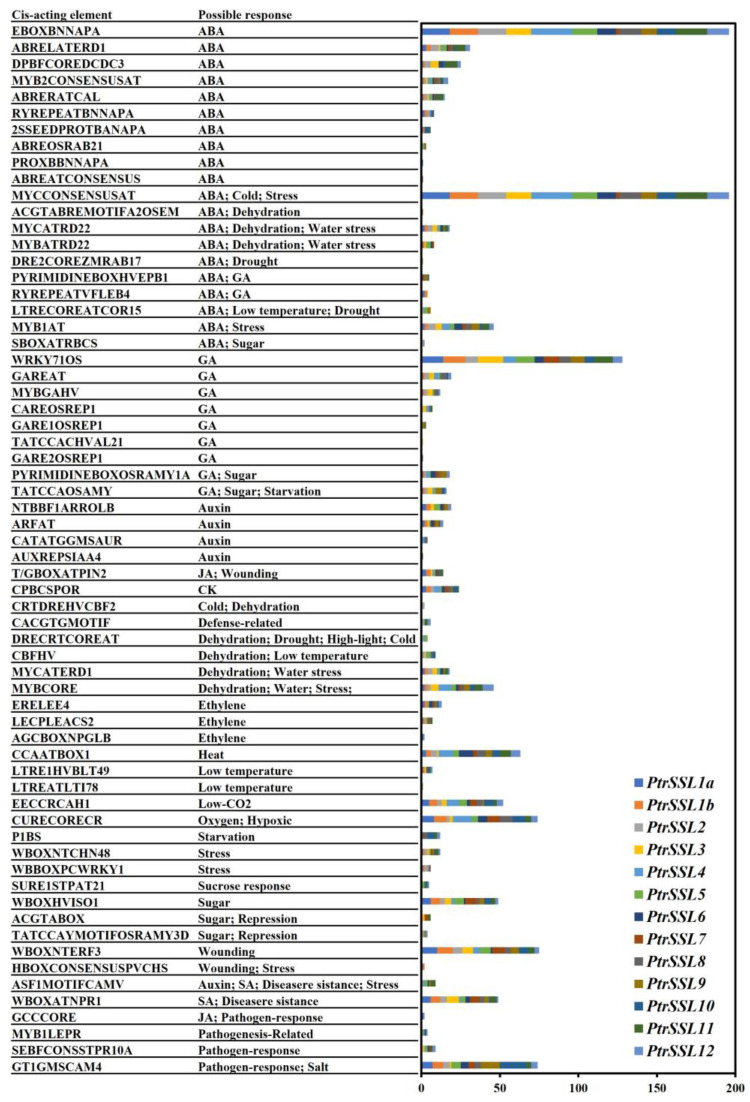
Cis-elements analysis of poplar *SSL* genes promoters. Different colors represent different genes. The element counts are shown in Supplementary Table S3.

Cis elements are specific DNA sequences located upstream of the gene coding sequence that can bind to regulatory proteins. We predicted the cis-elements in the 2000 bp upstream sequence of all *PtrSSL*s through PLACE [21]. As shown in Figure 5 and Supplementary Table S3, the elements associated with stress and hormonal responses cover a wide range of family members. A large number of cis elements associated with abiotic stresses were found in the promoter regions of most *PtrSSL*s, for example, in response to dehydration (ACGTABREMOTIFA2OSEM, MYCATRD22, MYBATRD22, etc.), water stress (MYCATRD22, MYBATRD22, MYCATERD1, etc.), drought (DRE2COREZMRAB17, LTRECOREATCOR15, DRECRTCOREAT, etc.), low temperature (LTRECOREATCOR15, LTRE1HVBLT49, LTREATLTI78, etc.), cold (CRTDREHVCBF2, DRECRTCOREAT, MYCCONSENSUSAT, etc.), stress (MYB1AT, MYBCORE, HBOXCONSENSUSPVCHS, etc.), and so on. Similarly, a large number of cis-elements associated with biotic stresses were found in most member promoter regions, such as disease-resistance (ASF1MOTIFCAMV, WBOXATNPR1), pathogen-response (GCCCORE, SEBFCONSSTPR10A, GT1GMSCAM4), and pathogenesis-related (MYB1LEPR) ones. A total of 41 phytohormone-related elements were identified, including abscisic acid response elements (EBOXBNNAPA, ABRELATERD1, DPBFCOREDCDC3, etc.), salicylic acid response elements (ASF1MOTIFCAMV, WBOXATNPR1), gibberellin response element (WRKY71OS, GAREAT, MYBGAHV, etc.), auxin response elements (NTBBF1ARROLB, ARFAT, CATATGGMSAUR, etc.), jasmonic acid response elements (T/GBOXATPIN2 and GCCCORE), and ethylene response elements (ERELEE4, LECPLEACS2, and AGCBOXNPGLB). In addition, each of the *PtrSSL*s’ promoters contained abundant ABA response elements, with *PtrSSL4* containing the largest number, i.e., 65, and *PtrSSL7* containing the lowest number, i.e., 15. All of these results point to the possible involvement of the *PtrSSL* gene in the plant response to hormones and biotic/abiotic stresses.

### 2.4. Expression Patterns of PtrSSLs in Roots, Stems, and Leaves

**Figure 6:**
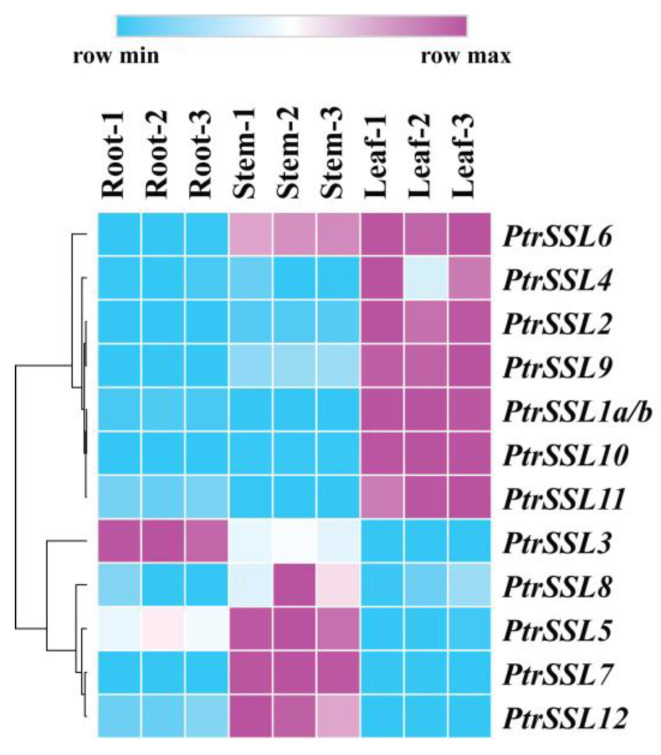
The expression patterns of *PtrSSL*s in roots, stems, and leaves. *PtrActin* was used as a reference gene. The expression of genes in root was set to 1. The data were processed using the 2^−ΔΔCt^ method.

To investigate the expression pattern of *PtrSSL*s in poplar, three tissues (root, stem, and leaf) were sampled from *Populus trichocarpa* for qPCR assays. The cluster analysis revealed that seven *PtrSSL*s exhibited high expression levels in leaves, among which *PtrSSL*6 was highly expressed in both stems and leaves, indicating their potential role in leaf function (Figure 6 and Supplementary Table S4). Additionally, four *PtrSSL*s, namely *PtrSSL*5, *PtrSSL*7, *PtrSSL*8, and *PtrSSL*12, exhibited high expression levels in stems, suggesting their involvement in stem function. Only *PtrSSL*3 showed high expression levels in roots, indicating its specific function in the roots. Overall, the different members of *PtrSSL*s have different expression patterns in roots, stems, and leaves, suggesting that the function of these genes may involve different biological processes.

### 2.5. Analysis of Upstream TF Regulation Network

**Figure 7:**
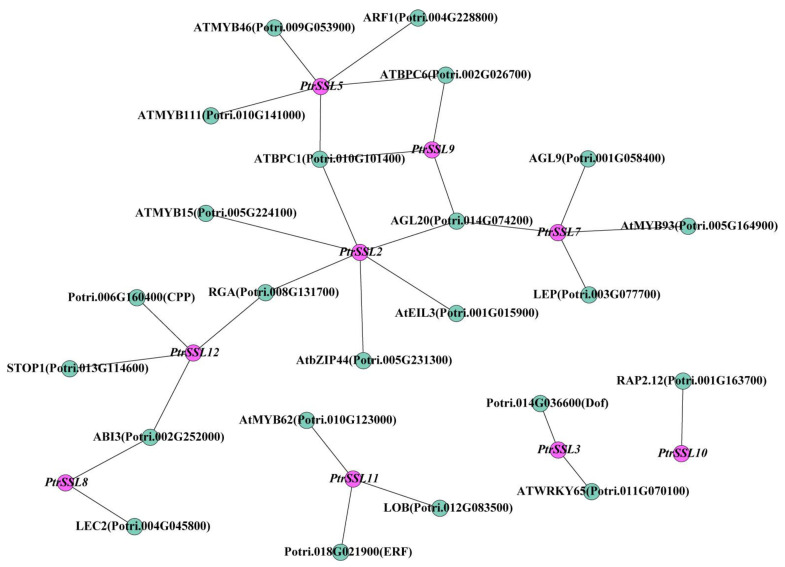
Transcriptional regulatory networks involving *PtrSSL*s. Pink nodes represent *PtrSSL*s, and green nodes represent potential regulators of *PtrSSL*s upstream.

In order to gain a better understanding of the underlying function of *PtrSSL*s, their upstream regulators were predicted by utilizing online websites and transcriptome data. The results show that the network consists of 9 *PtrSSL*s and 23 transcription factors (Figure 7). Based on previous studies, several of these TFs have been reported to be associated with plant adversity stress. For example, *ABI3* mediates dehydration-stress signaling in Arabidopsis through the regulation of a group of genes that play a role primarily during the stress-recovery phase [22]. *MtABI3* overexpression enhanced tolerance of transgenic *Medicago truncatula* to mannitol, drought, and salt stresses and induced the expression of adversity-related genes [23]. *AGL20/SOC1* can repress a broad array of genes that mediate abiotic stress responses in flowering induction [24]. Arabidopsis *BPC1/BPC2* positively regulates plant salt tolerance by repressing *GALS1* expression and β-1,4-galactan accumulation [25]. *MYB15* and *MYB46* are both from the MYB family, and one study found that *MYB15* is essential for basal immunity (PTI) in Chinese wild grape [26]. The *myb15* mutant plants show increased tolerance to freezing stress, whereas its overexpression reduces freezing tolerance [27]. *MYB46* likely functions as a disease-susceptibility modulator to *Botrytis cinerea* through the integration of cell wall remodeling and downstream activation of secondary lines of defense [28]. *MYB46* could enhance salt and osmotic stress tolerance in apple by directly activating stress-responsive signals [29]. In addition, *WRKY65* [30], *RGA* (*AtRGA1*) [31,32], and *STOP1* [33] have also been reported to respond to or participate in a variety of stresses.

### 2.6. RT-qPCR Validation of PtrSSLs under Different Stresses

**Figure 8:**
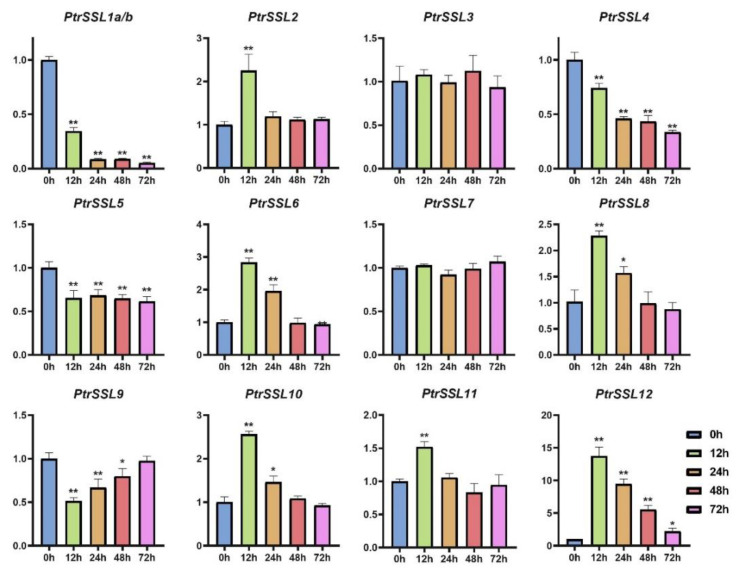
Expression patterns of *PtrSSL*s in response to NaCl. X-axis shows stress treatment time points, and Y-axis represents the relative expression level. The data were processed using the 2^−ΔΔCt^ method. Gene expression in 0 h was set to 1, and expression in the other time points was relative to it; *t*-test, * *p* < 0.05, and ** *p* < 0.01.

**Figure 9:**
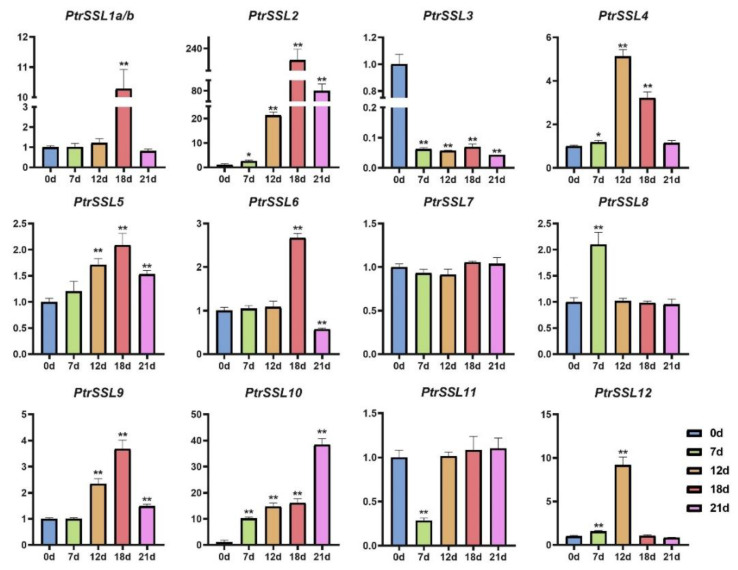
Expression patterns of *PtrSSL*s in response to drought. X-axis shows stress treatment time points, and Y-axis represents the relative expression level. The data were processed using the 2^−ΔΔCt^ method. Gene expression in 0 h was set to 1, and expression in the other time points was relative to it; *t*-test, * *p* < 0.05, and ** *p* < 0.01.

**Figure 10:**
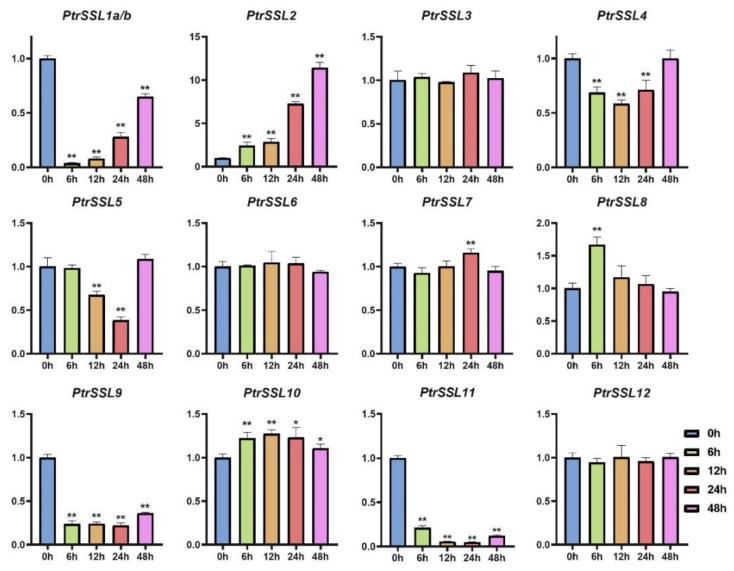
Expression patterns of *PtrSSL*s in response to leaf blight (*Alternaria alternata*). X-axis shows stress treatment time points, and Y-axis represents the relative expression level. The data were processed using the 2^−ΔΔCt^ method. Gene expression in 0 h was set to 1, and expression in the other time points was relative to it; *t*-test, * *p* < 0.05, and ** *p* < 0.01.

The results of the promoter analysis indicate that the promoter regions of *PtrSSL*s cover a large number of biotic/abiotic-stress-response elements, implying that they may have a positive response to biotic/abiotic stresses. To verify this, we subjected wild-type poplars to drought, salt, and leaf-blight stress and used RT-qPCR to quantify the expression patterns of *PtrSSL*s under different stresses (Supplementary Table S5). As shown in Figure 8, the expressions of six *PtrSSL*s were significantly upregulated in response to salt stress, namely *PtrSSL2*, *PtrSSL6*, *PtrSSL8*, *PtrSSL10*, *PtrSSL11*, and *PtrSSL12*. Four *PtrSSL*s were significantly downregulated after salt stress, namely *PtrSSL1a/b*, *PtrSSL4*, *PtrSSL5*, and *PtrSSL9*. Only the expression of *PtrSSL3* and *PtrSSL7* showed no significant change after salt stress. Then, as shown in Figure 9, only *PtrSSL7* showed no significant change in expression following drought stress, with most *PtrSSL*s (nine genes) demonstrating significant upregulation of expression in response to drought and a few *PtrSSL*s (*PtrSSL3* and *PtrSSL11*) showing significant downregulation of expression. The results after leaf-blight stress showed significant changes in the expression of most *PtrSSL*s (Figure 10), except for *PtrSSL3*, *PtrSSL6*, and *PtrSSL12*, which showed no response. Among them, *PtrSSL2*, *PtrSSL7*, *PtrSSL8*, and *PtrSSL10* showed significant upregulation of expression, while *PtrSSL1a/b*, *PtrSSL4*, *PtrSSL5*, *PtrSSL9*, and *PtrSSL11* were significantly downregulated. Overall, *PtrSSL*s have different expression patterns under different types of stress, and they may play important functions in plant resistance to biotic/abiotic stresses.

## 3. Discussion

Plants withstand complex and diverse environments by supervising a large number of stress-responsive and structural genes, engendering many physiological and metabolic processes [34]. The *SSL* gene family plays a key role in plant resistance to biotic/abiotic stresses, and although a growing number of *SSL* family members have been identified from a variety of plants, knowledge of their function is still restricted to a small number of plants, such as in Arabidopsis [31,32], *Catharanthus roseus* [14], and *Solanum lycopersicum* [19]. However, no relevant studies on the *SSL* family have been found in the poplar.

A total of 13, 15, and 22 *SSL* genes were obtained in *Populus trichocarpa*, *Arabidopsis thaliana*, and *Salix purpurea*, respectively, where the number of *AtSSL* genes that we obtained is consistent with previous reports [1,13], indicating that the results of our evolutionary analysis are stable. We found that *Populus trichocarpa* and *Salix purpurea* are more closely related evolutionarily than Arabidopsis by performing a phylogenetic analysis, probably because both are woody plants. The gene structure and motif analysis revealed that *PtrSSL*s in the same subgroup contained similar numbers of exons and motif species, which corroborated the phylogenetic tree distribution results. As we know, gene duplication, which is the main pattern of gene family expansion, includes tandem duplication and segmental duplication events [35,36,37]. The collinearity analysis and chromosome localization showed that most *PtrSSL*s were unevenly distributed across different chromosomes, with no tandem replication events occurring, while segmental replication events occurred in four pairs of *PtrSSL*s, suggesting that segmental replication plays an important role in the expansion of the poplar *SSL* family. The result of the cross-species collinearity analysis revealed that the largest number of *PtrSSL* collinear genes were present in *S. purpurea* and *E. grandis* probably because all three species are woody plants, implying that the evolution of *SSL*s may have diverged with the differentiation of herbaceous and woody plants. Overall, the bioinformatic analyses indicate that the *PtrSSL* gene family has similarities in phylogenetic and structural features to several species, suggesting that the function of *SSL* in these species may also be similar.

We found that the promoters of all *PtrSSL*s contained many biotic/abiotic-stress-response elements. Therefore, we subjected the plant material to different treatments, including salt, drought, and leaf blight fungus. The qPCR results for the three treatments showed that most *PtrSSL*s responded significantly to salt, drought, and leaf-blight stresses, suggesting that *PtrSSL*s may be key functional genes in the biotic/abiotic-stress-response pathway. By predicting the upstream TF regulatory network, we found that the response of *PtrSSL*s to stress may be induced by upstream TFs. For example, the qPCR results showed that *PtrSSL2* was able to respond to drought, salt, and leaf blight and that its upstream regulator, *RGA*, was able to respond to drought and salt stress in Arabidopsis [31,32], while the regulators *AGL20* [24], *ATBPC1* [25], and *ATMYB15* [26] were able to participate in drought, salt, and immunoregulatory processes in plants, respectively. It can hypothesize that the response of *PtrSSL2* to drought, salt, and leaf blight is induced by *RGA*, *AGL20*, *ATBPC1*, and *ATMYB15*. Also regulated by *ATBPC1* and *AGL20* in the TF regulatory network is *PtrSSL9*, a gene whose response to drought and salt stress may be induced by *ATBPC1* and *AGL20*. *MYB46* has an important function in plants’ resistance to disease and salt stress [28,29], and the altered expression of its target gene, *PtrSSL5*, during salt and leaf-blight stresses may be regulated by it. The expression of *PtrSSL12* was significantly upregulated in response to drought and salt stress, and it was regulated by *RGA*, *ABI3* [22,23], and *STOP1* [33] in the TF regulatory network; all three TFs were reported to be involved in drought- and salt-stress-related biological processes in plants [22,23,31,32,33], so the elevated expression of *PtrSSL12* may be activated by *RGA*, *ABI3*, and *STOP1*. Also likely to be activated by *ABI3* is *PtrSSL8*, whose expression was significantly increased in drought, salt, and leaf-blight stresses. It has been shown that *WRKY65* plays an important role in the early stages of drought stress [30], and in our TF regulatory network, *PtrSSL3* is predicted to be regulated by *WRKY65*, and the gene is significantly upregulated in expression after drought stress; this process may be induced by *WRKY65*. In conclusion, the results of our promoter analysis and gene expression following adversity stress indicate that the *PtrSSL* family may be one of the key functional gene families in the biotic/abiotic-stress-response pathway in plants.

## 4. Materials and Methods

### 4.1. Identification of SSLs in Poplar

Amino acid sequences of *Populus trichocarpa* (*Populus trichocarpa* v4.1), *Arabidopsis thaliana* (TAIR10), and *Salix purpurea* (*Salix purpurea* v5.1) SSLs were extracted from the Phytozome database (https://phytozome-next.jgi.doe.gov/, accessed on 12 March 2023), and the conserved structural domains Strictosidine synthase (PF03088) and Strictosidine synthase-like N-terminal (PF20067) were identified by Hidden Markov Model (HMM) profiling [38]. The two structured domains were obtained from the Pfam database (http://pfam.xfam.org/, 12 March 2023). The physical and chemical parameters of SSL proteins were calculated using the ExPASy website (http://web.expasy.org/protparam/, accessed on 12 March 2023).

### 4.2. Phylogenetic and Structural Analysis of PtrSSLs

Based on amino acid sequences from members of the *Populus trichocarpa*, *Arabidopsis thaliana*, and *Salix purpurea* SSL families, multiple sequence alignment was first performed using Clustal X [10]. Subsequently, MEGA X software [39,40] with the neighbor-joining method was used to construct a phylogenetic tree with the bootstrap value set to 10,000. MEME [41] was used to identify the motif compositions and distributions of *PtrSSL*s. All of those generated files were visualized using TBtools [42] and Itools software (https://itol.embl.de/, accessed on 13 March 2023).

### 4.3. Chromosomal Localization and Collinearity Analysis

*Populus trichocarpa* genomic data downloaded from the Phytozome database (*Populus trichocarpa* v4.1) were used to map each *PtrSSL* gene to its corresponding chromosomal location based on its positional information with TBtools [42]. MCScan X (Multicollinearity Scanning Toolkit) software [43] was used to determine their covariance relationships, which were visualized using TBtools v1.120 [42].

### 4.4. Cis-Acting Element Analysis

The sequence 2000 bp upstream of the transcription start site (TSS) of each *PtrSSL* was extracted from the Phytozome database. Cis-elements were predicted with PLACE [21] and visualized with TBtools.

### 4.5. Upstream TF Regulation Network Analysis

To predict the upstream regulators of *PtrSSL*s, we utilized the PlantRegMap website (http://plantregmap.gao-lab.org/go.php, accessed on 14 April 2023) and obtained the expression information of upstream genes from transcriptome data (SRP267437). We calculated the Pearson correlation coefficients between upstream genes and *PtrSSL*s and screened significantly related gene pairs, using a threshold of *p* ≤ 0.05. Finally, we plotted networks, using Cytoscape v3.3.0 [44].

### 4.6. Plant Treatments

The *Populus trichocarpa* clone Nisqually-1 was used in this study [45]. The plantlets were planted in humus soil and grown under a 16/8 h day/night photoperiod at 25 °C in the greenhouse. The different tissue samples were collected from 90-day-old poplar seedlings for a gene-expression analysis. To conduct the drought treatment, 90-day-old plants grown in the same environment were selected, and the water was withheld for varying durations. Similarly, 90-day-old plants were chosen for salt treatment and treated with a 200 mM NaCl solution for different periods, ranging from 0 to 72 h. Additionally, 90-day-old plants were sprayed with *Alternaria alternata* spore suspension (1.0 × 107 spores mL^−1^). The fungus was prepared as previously described [46,47,48]. The third-to-eighth functional leaves were harvested for RNA isolation. Plants treated with water were used as controls for all stress treatments. All samples had three biological replicates.

### 4.7. qRT-PCR Analysis

Total RNA was extracted using the Qiagen RNeasy Plant Mini Kit (QIAGEN, Hilden, Germany), and first-strand cDNA was synthesized using the PrimeScript™ RT reagent Kit with gDNA Eraser (TaKaRa, Beijing, China). Gene-expression patterns were identified using THUNDERBIRD^®^ Next SYBR^®^ qPCR Mix (TOYOBO, Osaka, Japan), and *PtrActin* was used as a reference gene for normalization. The 2^−ΔΔCT^ method was employed to analyze the relative expression changes of genes [49]. All of the qPCR reactions were conducted with three replicates. Standard errors and standard deviations were calculated from three replicates.

## 5. Conclusions

In this study, we identified 13 *SSL* genes from poplar, and the phylogenetic analysis revealed that these genes could be divided into four subgroups. The structure analysis and motif analysis showed that the gene structure and motif species of each subgroup member were similar. The results of the collinearity analysis indicated that *PtrSSL*s had more collinear genes in the woody plants *Salix purpurea* and *Eucalyptus grandis*, suggesting that the genes may have diverged, with the differentiation of herbaceous and woody plants. The chromosomal localization results revealed that the thirteen *PtrSSL* genes were unevenly distributed on seven chromosomes and one scaffold. Our analysis of cis-elements in promoters indicated that the promoters of *PtrSSL*s contain a large number of biotic/abiotic-stress-response elements and that these genes are likely to be involved in biotic/abiotic-stress responses. The RT-qPCR results indicated that 10, 11, and 9 *PtrSSL*s were able to respond to salt, drought, and leaf-blight stresses, respectively. In addition, we screened for multiple TF regulators in the upstream of *PtrSSL*s, such as *ATMYB46*, *ATMYB15*, *AGL20*, *STOP1*, *ATWRKY65*, and so on, which may act as activators/repressors of *PtrSSL*s in the process of plant resistance. This study provides a theoretical basis for the functional study of *SSL*s.

## Figures and Tables

**Table 1:** Basic information of *SSL* genes identified.

	Gene ID	Symbol	Length	Molecular Weight	Theoretical pI	Aliphatic Index	GRAVY
*Populus* *trichocarpa*	Potri.T015518	PtrSSL1b	357	39,445.26	7.49	89.52	−0.015
	Potri.017G027600	PtrSSL12	409	45,895.72	6.15	86.63	−0.238
	Potri.016G037900	PtrSSL9	377	41,011.22	7.09	96.4	−0.032
	Potri.016G037800	PtrSSL11	98	10,878.46	5.5	102.65	0.019
	Potri.016G037700	PtrSSL6	410	45,566.97	7.12	87.29	−0.162
	Potri.015G037700	PtrSSL7	335	36,270.25	9.14	91.37	0.018
	Potri.012G046200	PtrSSL3	315	34,126.71	9.4	86.1	0.037
	Potri.008G109966	PtrSSL1a	357	39,445.26	7.49	89.52	−0.015
	Potri.007G130700	PtrSSL8	406	45,861.89	6.68	89.26	−0.193
	Potri.006G140500	PtrSSL10	365	40,733.89	5.73	96.93	−0.044
	Potri.006G040900	PtrSSL2	388	42,747.89	9.38	90.49	−0.172
	Potri.005G099400	PtrSSL4	570	62,550.88	6.11	97.3	−0.015
	Potri.001G214500	PtrSSL5	391	43,983.39	6.48	84.5	−0.267
*Arabidopsis* *thaliana*	AT5G22020	AtSSL15	395	44,527.85	6.52	86.08	−0.159
	AT3G59530	AtSSL13	403	45,628.54	6.41	84.86	−0.227
	AT3G57030	AtSSL10	374	41,001.16	7.71	95.67	0.009
	AT3G57020	AtSSL9	370	41,457.8	6.56	88.19	−0.189
	AT3G57010	AtSSL8	376	41,980.07	5.78	83.62	−0.164
	AT3G51450	AtSSL7	371	41,124.39	5.4	104.29	0.07
	AT3G51440	AtSSL6	371	41,383.49	5.83	96.68	0.011
	AT3G51430	AtSSL5	371	41,641.9	6.2	95.36	−0.02
	AT3G51420	AtSSL4	370	41,595.67	5.65	96.16	0.054
	AT2G41300	AtSSL1	394	44,391.42	6.21	75.2	−0.403
	AT2G41290	AtSSL2	376	41,475.26	6.2	83.96	−0.145
	AT1G74020	AtSSL12	335	35,293.13	5.6	86.12	0.07
	AT1G74010	AtSSL14	325	34,184.1	8.27	86.98	0.155
	AT1G74000	AtSSL11	329	34,666.8	9.66	83.22	0.024
	AT1G08470	AtSSL3	390	44,036.55	8.11	84.95	−0.239
*Salix* *purpurea*	Sapur.T191900	SpuSSL6	304	33,213.88	8.98	93.98	0.118
	Sapur.T191200	SpuSSL7	324	34,854.57	7.58	91.2	0.151
	Sapur.T190300	SpuSSL11	262	28,228.92	6.58	94.16	0.029
	Sapur.15ZG033100	SpuSSL13	324	34,902.61	7.58	90	0.148
	Sapur.15WG054500	SpuSSL14	324	35,060.81	6.34	90.59	0.141
	Sapur.15WG054100	SpuSSL15	286	31,575.3	9.16	96.43	0.173
	Sapur.15WG052000	SpuSSL16	324	34,932.64	7.58	90	0.135
	Sapur.15WG051300	SpuSSL17	288	30,907.06	7.58	87.05	0.115
	Sapur.15WG051000	SpuSSL18	335	36,203.24	8.62	91.43	0.139
	Sapur.15WG050600	SpuSSL3	162	17,593.19	4.69	88.02	0.349
	Sapur.15WG050400	SpuSSL19	324	34,978.71	7.58	90	0.158
	Sapur.15WG050300	SpuSSL20	200	22,144.36	9.8	94.65	−0.148
	Sapur.15WG049100	SpuSSL21	262	28,296.15	8.76	96.76	0.061
	Sapur.15WG048500	SpuSSL22	271	29,352.2	6.58	93.54	0.029
	Sapur.017G016600	SpuSSL12	406	46,029.86	6.33	86.13	−0.234
	Sapur.016G034000	SpuSSL9	374	40,947.06	6.52	97.46	−0.034
	Sapur.008G088100	SpuSSL1	357	39,231.86	5.63	93.89	−0.023
	Sapur.007G116200	SpuSSL8	406	45,795.78	6.65	90.44	−0.166
	Sapur.006G115900	SpuSSL10	239	26,500.28	5.05	91.38	−0.055
	Sapur.006G030000	SpuSSL2	340	37,236.23	8.16	80.65	−0.222
	Sapur.005G078900	SpuSSL4	409	45,329.69	5.72	94.38	−0.128
	Sapur.004G165500	SpuSSL5	391	43,758.23	6.56	85.5	−0.204

**Table 2:** The Ka/Ks ratios of duplication for *PtrSSL*s.

Duplicated Gene Pairs	Ka	Ks	Ka/Ks	The Length of Homologous Fragment (bp)	Homology/%	Duplication Date (MYA)
PtrSSL1a-PtrSSL1b	0	0	0	1074	100	0
PtrSSL2-PtrSSL6	0.09842	0.346721	0.283861	1233	86.93	11.55735
PtrSSL3-PtrSSL7	0.187272	0.481373	0.389037	1008	80.65	16.04577
PtrSSL8-PtrSSL12	0.032732	0.265984	0.123062	1221	92.38	8.866147

## Data Availability

Not applicable.

## Supplementary Materials

The supporting information can be downloaded at https://www.mdpi.com/article/10.3390/ijms241210117/s1.
